# Lensless Photoluminescence Hyperspectral Camera Employing Random Speckle Patterns

**DOI:** 10.1038/s41598-017-14443-4

**Published:** 2017-11-10

**Authors:** Karel Žídek, Ondřej Denk, Jiří Hlubuček

**Affiliations:** Regional Centre for Special Optics and Optoelectronic Systems (TOPTEC), Institute of Plasma Physics, Academy of Sciences of the Czech Republic, Za Slovankou 1782/3, 182 00 Prague 8, Czech Republic

## Abstract

We propose and demonstrate a spectrally-resolved photoluminescence imaging setup based on the so-called single pixel camera – a technique of compressive sensing, which enables imaging by using a single-pixel photodetector. The method relies on encoding an image by a series of random patterns. In our approach, the image encoding was maintained via laser speckle patterns generated by an excitation laser beam scattered on a diffusor. By using a spectrometer as the single-pixel detector we attained a realization of a spectrally-resolved photoluminescence camera with unmatched simplicity. We present reconstructed hyperspectral images of several model scenes. We also discuss parameters affecting the imaging quality, such as the correlation degree of speckle patterns, pattern fineness, and number of datapoints. Finally, we compare the presented technique to hyperspectral imaging using sample scanning. The presented method enables photoluminescence imaging for a broad range of coherent excitation sources and detection spectral areas.

## Introduction

Photoluminescence (PL) emitted from a material provides an immense amount of information about the emitting matter. It can disclose the degree of molecular aggregation^1^, defects in materials^2^, reveal the size distribution of nanocrystals^3^, etc. For these reasons the PL measurement has become a standard means of spectroscopic sample characterization. PL imaging shifts the abilities of this method even further by the possibility to visualize and map PL characteristics in a sample^4^.

PL imaging in the visible spectral range is nowadays a well mastered task solved by standard hyperspectral imaging approaches^5–7^ such as variable spectral filters and push broom or whisk broom scanners. However, the task becomes more complicated in the infrared (IR) region due to the difficulty of adjustment of the IR optics, as well as the need for an expensive two-dimensional sensor and IR imaging elements^8,9^. A solution bypassing the difficulties is to focus the PL excitation to a tight spot and carry out imaging by scanning a sample, i.e. by shifting the excitation spot, and detecting the PL intensity by a single-pixel detector (photodiode, photomultiplier, spectrometer). However, sample scanning can become problematic for samples placed in a bulky device (cryostat, high-power magnets, etc.). Moreover, such an approach is highly time consuming. For instance, for a 128 × 128 pixel image with 1s PL detection integration time, the total measurement time reaches 4.5 hours.

Here we propose and demonstrate a simple lensless hyperspectral PL camera that allows PL imaging by using a single-pixel detector – a spectrometer providing the camera with spectral resolution^10,11^. Our approach makes it possible to decrease the number of measurements, compared to sample scanning, to about 20% of the image pixel number, while keeping the primary image information. The abovementioned example measurement (128 × 128 pixel, 1s acquisition) can therefore be carried out in about one hour. Moreover, we can achieve the imaging using only two optical elements – a transmissive or reflective diffusor and a beam splitting wedge or any equivalent element. Hence the method can be applied to a broad range of coherent excitation sources and PL detection ranges.

The method is based on compressed sensing^12,13^, namely the so-called single-pixel camera^14–16^. Compressed sensing is a novel approach to data acquisition used extensively in imaging. It involves encoding a piece of information, for instance, an image, by a random or a pseudo-random pattern and reconstructing it using computer algorithms. We refer the reader to a number of introductory and review articles for more details^13,17–19^. In our measurements we employ the so-called single-pixel camera concept^14^ where an image is encoded by a series of random patterns and the total intensity of light emitted from the sample, is detected and used for the image reconstruction.

Unlike in the standard single-pixel camera design, where imaging and relay optics are needed^16,20^, here we encode the detected scene by a random speckle pattern arising via an interference of many wavefronts with random phases. A similar concept was used previously employing sophisticated optical devices (e.g. a digital micromirror device)^21^. To generate the speckle pattern, we, instead, employ a simple approach using a moving diffusor, which can be easily incorporated into any laser-excited PL detection. This leads to a PL imaging setup of unmatched simplicity.

In this article we present a hyperspectral PL camera and demonstrate its performance on a solution of Rhodamine 6G. We address parameters affecting the camera function, such as speckle patterns correlation or fineness. Finally, we discuss the possible range of applicable measurements.

## Imaging System

**Figure 1:**
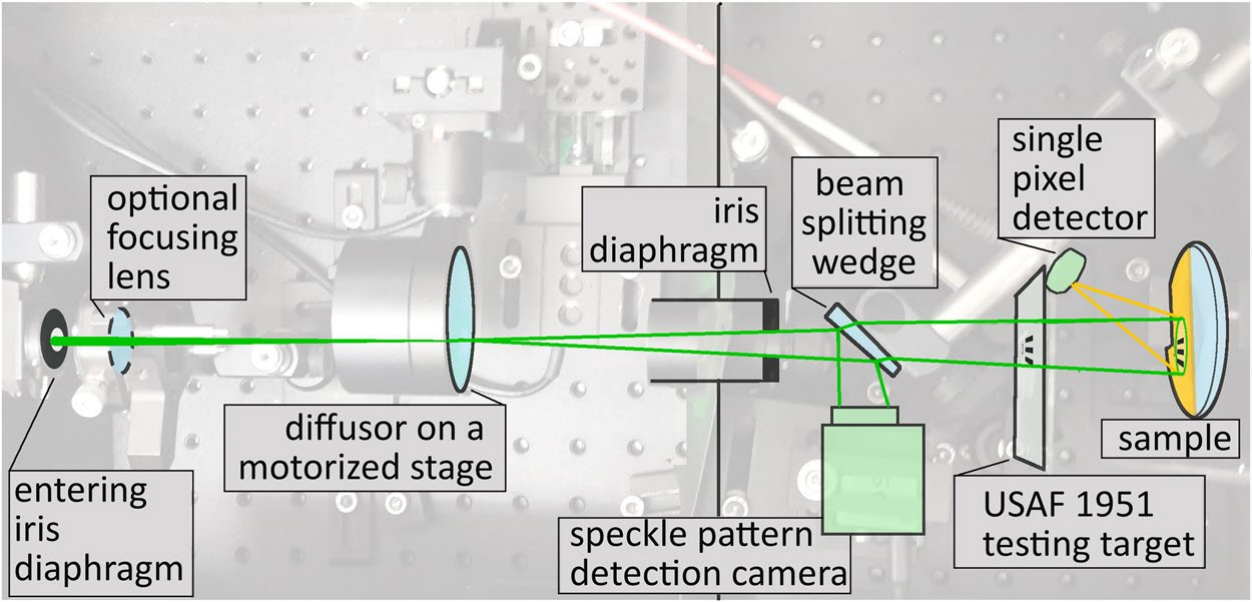
Photograph of the experimental setup overlaid with a simple scheme. The laser focusing lens can be used to modify the speckle pattern properties (see text for details). USAF 1951 testing target was used to verify the resolution of the imaging setup. The single pixel detector in the presented results was a fiber-coupled spectrometer.

Our experimental setup (depicted in Fig. 1) was based on a standard PL measurement setup. The entering excitation laser beam was used to generate a speckle pattern. The beam can be optionally focused by a lens to adjust the mean size of speckles, as we discuss later. The lens is, however, not needed for the setup operation. Consequently, a diffusor on a XY-motorized stage (Thorlabs) was placed in the laser beam. The diffusor served as a medium for obtaining a speckle pattern. The speckle pattern was reduced via an iris diaphragm into a narrow cone of patterned light, which was used for the sample excitation. A small fraction of the beam intensity was reflected from a wedge onto a reference CMOS chip (IDS camera). The wedge was used in order to avoid interference of multiple beam reflections on the camera.

The sample PL emission can be collected efficiently by placing the sample into an integrating sphere, by using a parabolic mirror, or it can be collected by a lens. However, we put emphasis on the simplicity of the demonstrated experimental approach. Therefore, the emitted PL was collected by an optical fiber (1 mm core), which was placed in the sample proximity. The detector distance was set so that the light from the entire illuminated area was evenly collected. This was adjusted prior to each experiment by using back-propagating light (Ocean Optics, LS-1 lamp) through the detecting optical fiber. The cone of the back-propagating light illuminating the sample determines the area from which the emission is collected.

We employed Rhodamine 6G (rh6G) in a flat cuvette as a testing sample. A laser source at 532 nm (frequency doubled Nd:YAG laser operating at 1064 nm) at a moderate excitation power of 3 mW was used to generate a speckle pattern. This assured that the PL emission remained in the linear regime. The testing was carried out by using a positive USAF 1951 resolution target or by imaging the edges of the rh6G solution. Due to the diverging speckle pattern, the resulting USAF pattern shadow was scaled up approx. 1.25-times. The illuminated sample area, which at the same time defines the imaging area, was a circle approx. 4 mm in diameter. The light emitted from the sample was analyzed via a spectrometer (Ocean Optics, Flame).

The single-pixel camera concept relies on detecting a series of total intensities for known encoded random-pattern illuminations. We obtained the total intensity value by selecting a spectral range to reconstruct. For each measurement, the PL spectrum in the range was averaged, thus providing the total intensity. The random encoding pattern was obtained as a speckle pattern image on the reference CMOS chip. Since the experimental setup was based on widely available components (laser source, camera, detectors), the signals undergo a minor drift of the ratio between the speckle pattern intensity and the detected intensity. The main source of the drift lies in the reference CMOS chip heating up during the measurement. The drift was corrected by dividing the variation in the total intensity smoothed in a long-term range of datapoints (Savitzky-Golay smoothing, 350-point range).

The reconstruction of an image *u* with *N* pixels (e.g. N = 4096 for 64×64 image) from *M* measurements was carried out using TVAL3 algorithm^22^ minimizing the expression:

1 $${\min }_{u\in {R}^{n}}\sum _{i}{\Vert {D}_{i}u\Vert }_{1}+\frac{\mu }{2}{\Vert Au-b\Vert }_{2}^{2},{\rm{s}}{\rm{.t}}.\,u\ge 0.$$

*D* stands for differentiation along x- and y- axis. *A* represents the so-called measurement matrix $N\times M$, which is composed of *M* columns, each representing one speckle pattern with *N* pixels. Finally, *b* stands for the vector of total intensities (*M* elements). A detailed description of the reconstruction procedure and the used parameters can be found in the supplementary information.

## Results and Discussion

### Speckle Patterns Correlation

**Figure 2:**
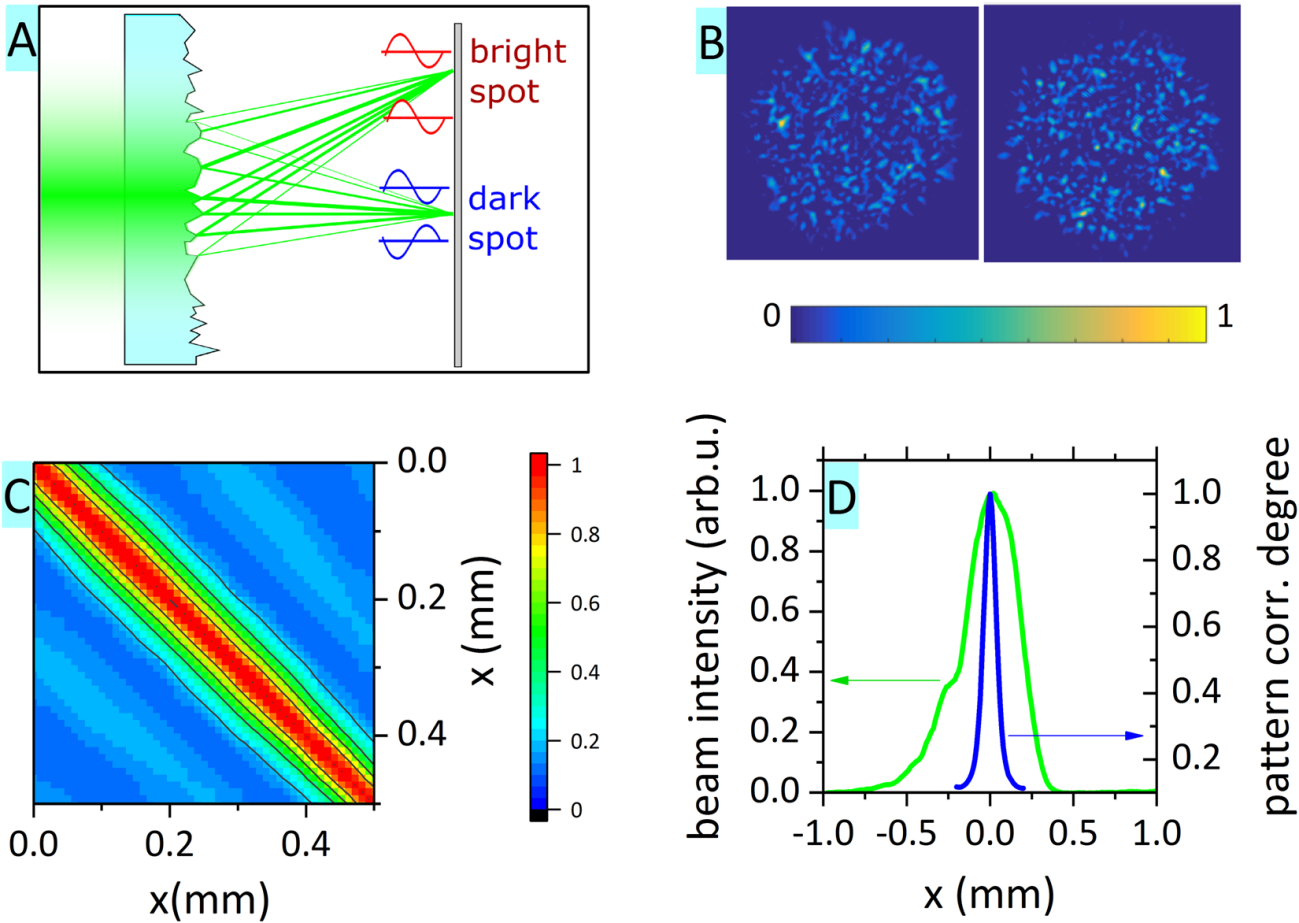
(**A**) Scheme of speckle pattern origin – depending on the random phase, the amplitudes add up (bright spot) or subtract (dark spot); (**B**) Two examples of fine speckle patterns used in the experiments; (**C**) Correlation degree (see text for details) between speckle patterns acquired at different diffusor positions; (**D**) Comparison of beam profile (green line) and averaged correlation degree between neighboring patterns (blue line).

A laser speckle pattern is a random intensity pattern resulting from the interference of many wavefronts arising due to multiple scattering of coherent light (see Fig. 2A,B)^23^. First, we focused on ensuring that the acquired random patterns are not correlated with each other. This satisfies the need for incoherence in the measurement matrix *A* in the compressed sensing theory^13^.

By shifting the diffusor by a small step and acquiring a speckle pattern for each position, we obtained a set of images. The correlation between two images *I* and *J* can be evaluated based on the following expression, where σ_I_ denotes the standard deviation of the image I:

2 $$C(I,J)=\sum _{k,l}\frac{I(k,l)-\langle I\rangle }{{\sigma }_{I}}\frac{J(k,l)-\langle J\rangle }{{\sigma }_{J}}.$$

The expression is also similar to the calculation of the so-called mutual coherence of the sensing matrix *A*, where each speckle pattern represents one column in the matrix. A lower mutual coherence of a matrix ensures a good information reconstruction under a low number of measurements^17^.

We calculated the degree of correlation for a series of diffusor horizontal shifts (denoted as x-axis) – see Fig. 2C. The results show that the neighboring patterns were highly correlated due to the so-called memory effect^24^ and the correlation degree rapidly decreased on a scale significantly below the beam FWHM (beam FWHM 0.36 mm vs. correlation degree FWHM 0.1 mm, see Fig. 2D). The loss of correlation depends on the typical size of the random features (scattering sites) on the diffusor, as well as on the decrease in the overlap between the beams. The presented measurement therefore provided us with the information that the diffusor shift along the x-axis of 0.15 mm or greater ensures obtaining a sequence of uncorrelated random patterns. The situation was analogous for the y-axis, where the beam FWHM and correlation degree FWHM reached 0.4 mm and 0.1 mm, respectively.

### Image Reconstruction

**Figure 3:**
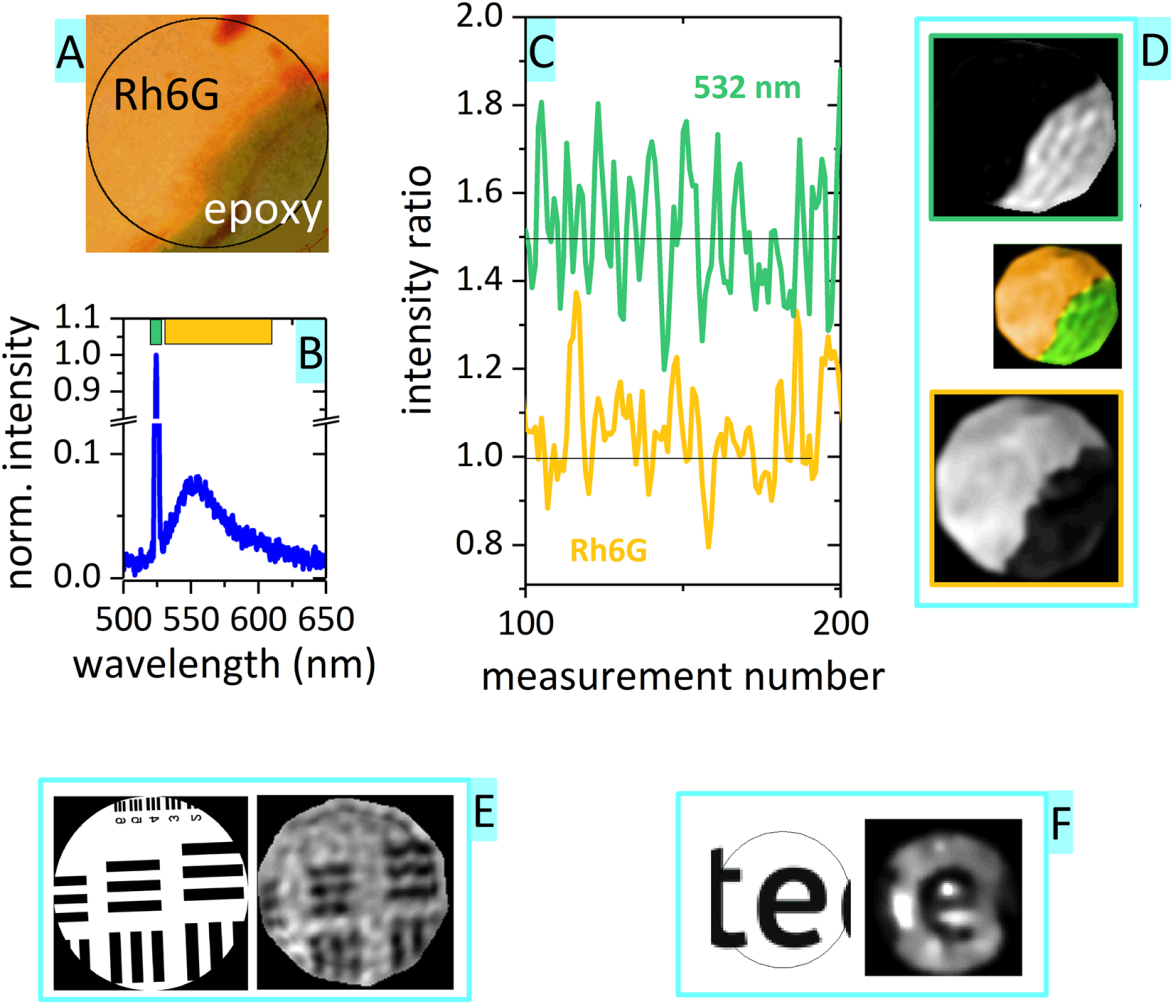
Examples of reconstructed images. (**A**) Scene of an interface between Rhodamine 6 G and scattering epoxy glue (black circle denotes the illuminated area); (**B**) Example of a spectrum detected during the measurement (normalized), color bars at the top denote the spectral ranges used for reconstruction in panel C; (**C**) Example of intensity points from the two spectral ranges (the intensity points were divided by the total speckle pattern intensity); (**D**) Reconstructed images (image: 128 × 128 pixels, 6200 datapoints, *M*/*N* = 39%) for the two spectral ranges (grayscale images) and merged image (orange-green image); (**E**,**F**) Lensless imaging, left side – original image, right side – reconstructed image; (**E**) A shadow of a fine USAF pattern on rh6G (image: 128 × 128 pixels, 4000 datapoints, *M*/*N* = 24%); F) Scattered laser light (image: 64 × 64 pixels, 680 datapoints, *M*/*N* = 17%).

The acquired knowledge was used to generate a series of measurements with the hyperspectral camera. As an example we present a measurement of an interface between PL-emitting rh6G and a scattering layer of epoxy glue (see Fig. 3A). Illumination of the indicated circle (black circle in Fig. 3A) led to a spectrum (see Fig. 3B) with a pronounced laser peak at 532 nm (scattered laser light) and the rh6G PL emission (540–650 nm spectral band). We averaged the detected intensities in the two spectral ranges 528–536 nm and 540–650 nm (see color bars in panel B) and obtained two sets of intensity values for each measurement (see Fig. 3C). The intensity datapoints together with the speckle pattern images were finally used to reconstruct the original image based on eq. (1), leading to a different image (Fig. 3D) for the PL band (bottom panel) and the scattering band (top panel). To provide a better comparison, we also superpose the results into a two-color reconstruction (middle image in panel D), proving that the reconstructed image corresponds very well with the original scene. In addition, we provide two examples of lensless imaging. One is of a fine USAF pattern shadow on rh6G (Fig. 3E) and the other of a scattered laser beam on a text (Fig. 3F).

### Imaging Parameters

Unlike in the standard implementations of a single pixel camera, speckle patterns can be very easily adjusted for the desired measurement area (by adjusting the pinhole) and pattern fineness (by focusing the laser beam). The mean speckle size for wavelength λ can be approximated in the far-field regime as $\frac{\lambda z}{d}$, i.e. the size is determined as a combination of the laser beam width *d* and the diffusor-sample distance *z*. Both parameters can be used for the speckle pattern fineness adjustment^23^. The fineness of a speckle pattern *I* can be quantified by calculating the pattern autocorrelation^25^:

3 $$c(p,q)=\sum _{m,n}I(m,n).I(m-p,n-q)$$

The autocorrelation features a pronounced central peak with width proportional to the mean speckle size.

**Figure 4:**
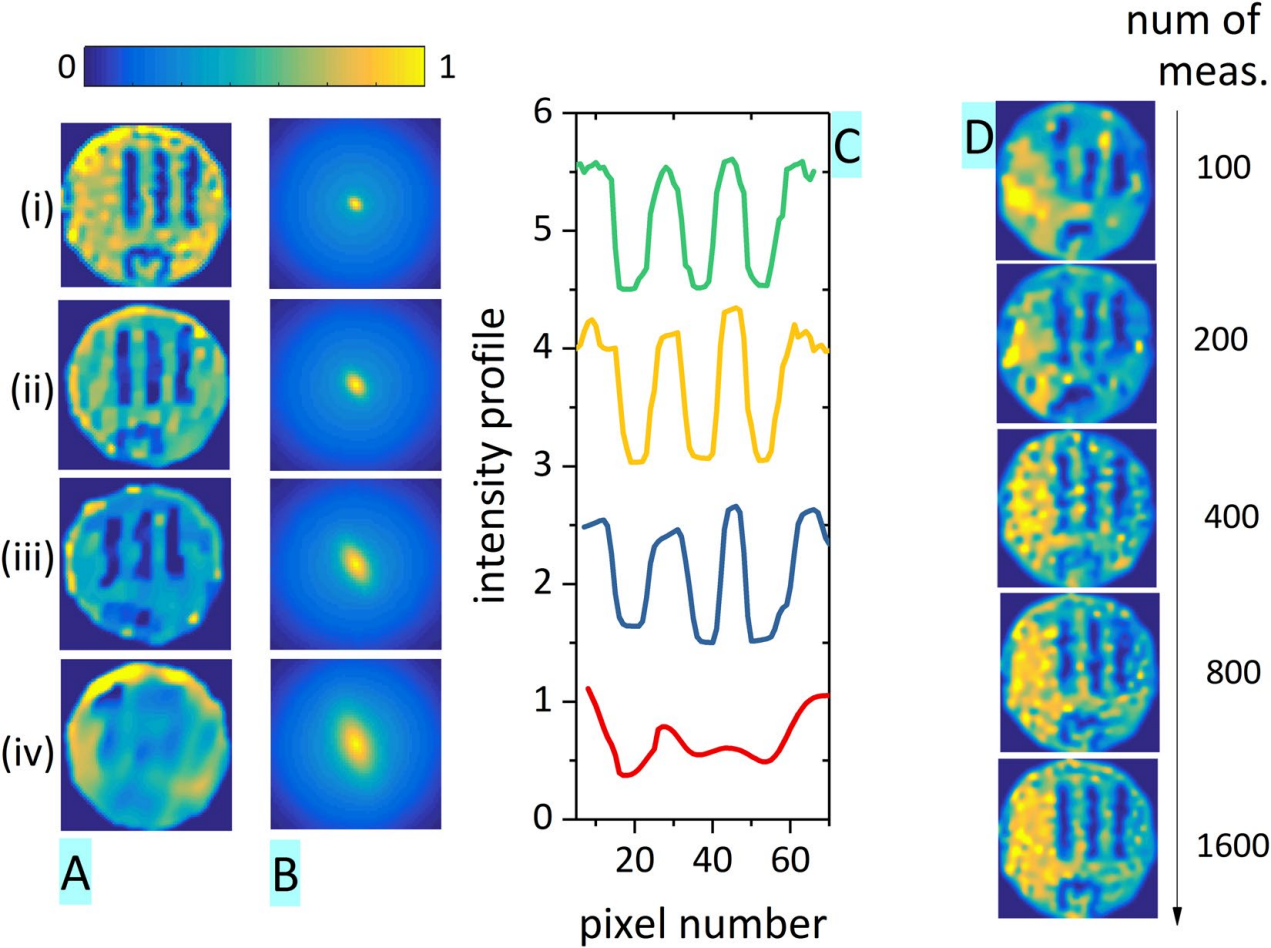
Reconstruction of a shadow of a part of USAF 1951 in Rhodamine 6G emission. (**A**) A reconstructed image for 4 different mean speckle sizes (*M* = 1500 measurements, 96 × 96 pixel image, *M*/*N* = 16%); (**B**) The mean autocorrelation of all speckle patterns; (**C**) Cross-section of three vertical target lines (averaged values from lines 50–65) for the 4 reconstructed images; (**D**) The same scene (USAF target shadow, speckle mean width σ_x_ = 3.1 pixel, σ_y_ = 3.2 pixel) reconstructed from an increasing number of datapoints, *M*/*N* = 1%, 2%, 4%, 9%, and 17%, respectively.

We investigated the relation between the mean speckle size and the setup resolution. The same testing image – a shadow of USAF resolution etalon on rh6G – was used to acquire a series of measurements with four different laser spot sizes. The variation in the spot size leads to four different degrees of fineness of the patterns, which can be evaluated based on the mean autocorrelation of all patterns (see Fig. 4B). Namely, we determined the root-mean-square width σ by fitting the horizontal and the vertical central peak cut with the Gauss function. The resulting σ values are listed in Table 1.

**Table 1:** Summary of the resulting root-mean-square widths of the speckle autocorrelation peak and image point spread function (PSF) estimate for the four measurements in Fig. 4A,B. For the measurement (iv) the PSF values could not be recovered.

meas.	speckle autocorr.		image PSF estimate	
	σ_x_	σ_y_	σ_x_	σ_y_
	[pixel]	[pixel]	[pixel]	[pixel]
i	3.1	3.2	1.7	2.0
ii	4.3	4.7	2.3	3.7
iii	5.4	6.5	3.4	4.7
iv	6.3	7.8	—	—

Imaging of the same testing scene with increasing mean speckle size led to a decrease in the reconstructed image resolution (see Fig. 4A). In order to illustrate this effect, we extracted for each case a horizontal cut through the three vertical lines in the USAF target (Fig. 4C). With the increasing speckle size the line edges become gradually less pronounced.

Furthermore, in order to quantitatively evaluate the resolution of the image reconstruction, we carried out a calculation, where the original image of the USAF target ${u}_{orig}$ was convoluted with a Gaussian point spread function (PSF) $P({\sigma }_{{x}},{\sigma }_{{y}})$ and compared with the reconstructed image *u*_*rec*_. The PSF root-mean-square widths *σ*_*x*_ and *σ*_*y*_ were optimized with respect to the minimum difference between the original and the reconstructed images. The resulting estimates of the PSF widths are listed in Table 1.

In general, we observe that the image PSF width reached about 50% of the speckle autocorrelation peak width. The results therefore confirm that the resolution is set by the speckle autocorrelation width. Interestingly, even though speckle patterns feature a broad distribution of speckle sizes, the fine details cannot be recovered by a series of coarse speckle patterns, in spite of using a high number of acquisitions reaching 100% of the image pixel count. The mean speckle size therefore represents the native resolution of the system that cannot be easily overcome.

Analogously to other single pixel camera techniques, each measurement here also carries a piece of information about the whole scene. The image can therefore be reconstructed based on a small number of datapoints (Fig. 4D). Nevertheless, a significant improvement is achieved by increasing the datapoint count. In Fig. 4D a reasonable reconstruction can be achieved for *M* = 1600 datapoints (*N* = 96 × 96 pixel image, *M/N* = 18%, detector noise std. dev. of 6%). It is, however, worth noting that in the case of the speckle-based single-pixel camera the resolution is dominantly affected by the mean speckle size and, therefore, the *M/N* ratio is, in fact, higher.

### Limitations of the Imaging Parameters

An important consideration for every imaging system are the limits to achievable spatial and spectral resolution. We will first turn to spectral resolution, which is provided in our case by a spectrometer. In principle the camera resolution is only limited by the resolution of the spectrometer itself. For the wavelengths where the typical detected signal exceeds 10^4^ counts (maximum 65536 counts) the reconstruction can be carried out from a single spectral datapoint. This is, however, often not the case for a reasonable integration time (1s or below) and a weak PL signal. There the realistic limitation of the system is set by the signal-to-noise ratio. Since the reconstruction discrepancy ${\Vert {u}_{rec}-{u}_{orig}\Vert }_{2}$ is proportional to the noise level^17^, it is advisable to average a spectral range, thus reducing the noise level by a square root of the number of the averaged spectral datapoints.

The spatial resolution of the imaging setup is determined by the speckle pattern fineness, which can be improved by reducing the sample-diffusor distance *z* or by increasing the laser spot diameter on the diffusor. This approach is, however, not valid for short distances *z*, where the speckle pattern rapidly changes with the distance from the diffusor (near-field speckle pattern). It is therefore not a straightforward task to assess the best obtainable value and this issue will be addressed in our future work. Nevertheless, the best real resolution obtained so far in the presented setup was about 20 μm. This value is based on an image of a single scattering site on a luminophore.

### Comparison to the sample scanning

It is worth comparing the setup to the approach where an excitation spot is focused and the sample is scanned in the XY manner to acquire the PL map. Both experiments provide PL hyperspectral imaging by using a single-pixel detector.

Firstly, sample scanning requires motorized stages with well-defined steps and a tightly-focused laser beam – both being determinants of the setup spatial resolution. On the contrary, the presented setup relies on shifting a diffusor, which can be performed randomly – without the necessity to control the exact diffusor position. Measurements with a very short acquisition time can be, for instance, acquired with a continuously-moving motorized stage, without any need to track the actual stage position.

Secondly, the mode of illumination is different for each method. For sample scanning, the sample area is illuminated, point-by-point, with a high-intensity excitation focused on a small fraction of the sample. In contrast, the presented approach continuously illuminates the entire imaging area. Nevertheless, to reach a similar detected PL intensity a low-intensity excitation is sufficient here. The difference has consequences for the samples which suffer from photobleaching. The samples undergoing photobleaching only above a certain light intensity threshold may benefit from using the excitation pattern. On the contrary, photobleaching with a low threshold would lead to a continuously decreasing total intensity. A possible way to abate the issue is to compensate for the long-term drift in the intensity values.

Finally, as we stated in the introduction, by involving compressive imaging, we can carry out the same hyperspectral imaging with a reduced number of measurements and use about 20–40% of datapoints compared to sample scanning.

## Conclusion

We have presented a simple experimental technique able to implement a PL hyperspectral camera by extending a standard PL measurement setup with a shifting diffusor. This approach is very versatile as far as PL detection and imaging parameters, such as imaged area or image resolution, are concerned. The presented experiments were carried out using a spectrometer as a single-pixel detector. However, by replacing the spectrometer with an IR detector, the same setup can serve for PL imaging in the IR region.

Moreover, the excitation pattern is prepared solely based on diffusor and reference imaging. For this reason, the presented setup can be used for excitation of coherent sources in the deep UV region, where a reflecting optical chopper with a luminophore-covered 2D sensor can serve as a pattern reference, or in the IR region, where an up-converting layer can provide the same information.

The presented setup can therefore provide a solution to a broad range of experimentally difficult cases requiring PL imaging.

## Electronic supplementary material

Supplementary Information
