# Supplementary material for: Lensless Photoluminescence Hyperspectral Camera Employing Random Speckle Patterns
Source: Sci Rep. 2017 Nov 10;7:15309. doi: 10.1038/s41598-017-14443-4 (PMC5681664; doi:10.1038/s41598-017-14443-4)
Supplement: Supplementary file 1 — Supplementary Information [file 41598_2017_14443_MOESM1_ESM.pdf]

Supplementary information

Lensless Photoluminescence Hyperspectral Camera Employing Random Speckle Patterns

Karel Žídek, Ondřej Denk, Jiří Hlubuček

Regional Centre for Special Optics and Optoelectronic Systems (TOPTEC), Institute of Plasma Physics, Academy of Sciences of the Czech Republic, Za Slovankou 1782/3, 182 00 Prague 8, Czech Republic

Image reconstruction procedure

Since the used speckle patterns illuminated a circular area on the rectangular 2D matrix, we firstly calculated a mask restricting the area of action. This was done by averaging all acquired speckle patterns, normalizing the average image to $\langle 0,1 \rangle$ and selecting the pixels with value above 0.02 – see Figure S1.

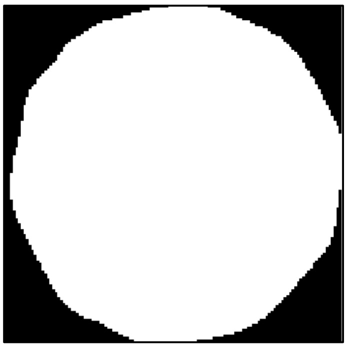

Figure S1. Example of a mask dividing the area of action (white) from the inactive part (black).

Secondly, we calculated a ratio between the total speckle pattern intensity and the intensity detected by the single-pixel detector. For a high number of random patterns the ratio is very unlikely to show long-range fluctuations, which was however commonly observed due to drift in the spectrometer and CMOS camera response. The drift was corrected by calculating a smoothed curve via Savitzky-Golay smoothing (the best results obtained for 350 datapoint range) and the total intensities were corrected for the the drift – see Figure S2. The optimum datapoint range was tested for the used experimental configuration and it is expected to vary based on the used detectors.

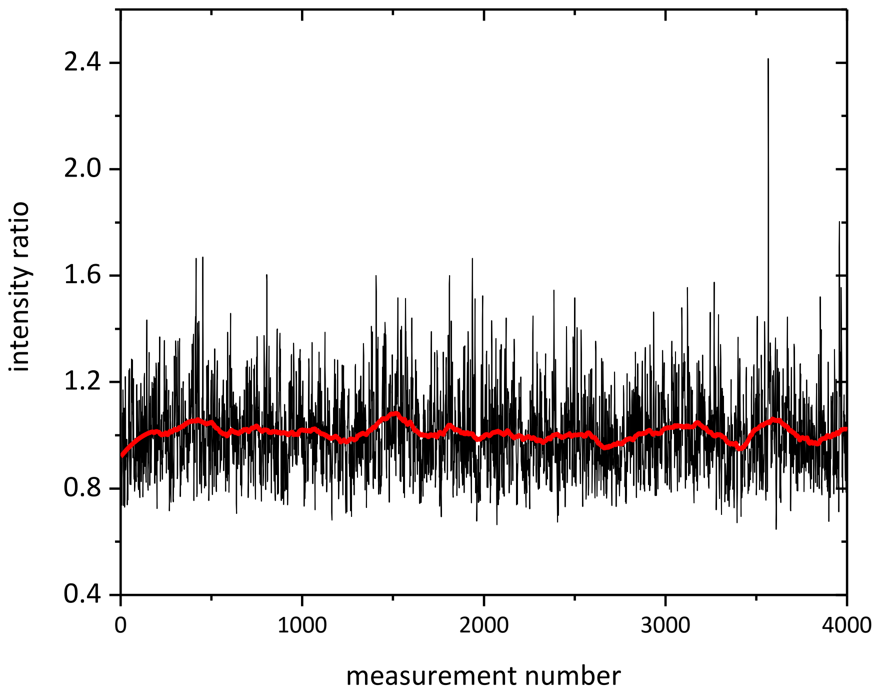

Figure S2. Ratio between the total speckle pattern intensity (CMOS chip data) and the recorded intensity (spectrometer data) – black line. Red line - a smoothed curve (Savitzky-Golay smoothing, 2nd order, 350 points).

An initial guess g for a particular pixel $g(n_1, n_2)$ was calculated based on correlation degree between the speckle patterns S for the particular image pixel $S(n_1, n_2, 1..M)$ and total intensity $b(1..M)$:

$$g(n_1, n_2) = \sum_m S(n_1, n_2, m) \, . [b(m) - \langle b \rangle] \quad . \tag{S1}$$

In the resulting initial guess (see Figure 3), all values below zero were set to zero. The initial guess reduces the number of iterations required to reconstruct the image, however, the reconstruction is robust and it can be initiated for instance by using the mask depicted in Figure S1. Images were reconstructed via TVAL3 algorithm[1] using implicit parameters (see reference for details), beside the values of μ and β, which were varied to obtain the best reconstruction for the particular case ($2^{7\text{-}10}$ and $2^{5\text{-}9}$, respectively).

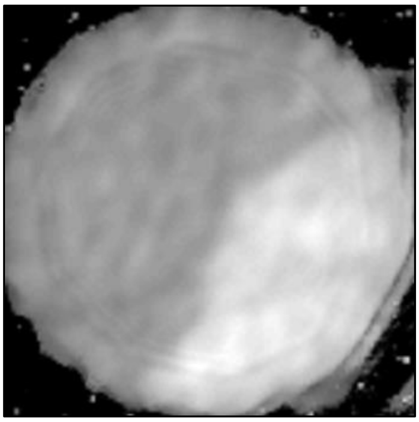

Figure S3. Initial guess of a reconstructed image - see Figure 3D in the article (top panel) for the final reconstruction.

References

1. Li, C., Yin, W. & Zhang, Y. User's guide for TVAL3: TV minimization by augmented lagrangian and alternating direction algorithms. *CAAM Rep.* **20,** 46–47 (2009).
